# A goal function approach to remodeling of arteries uncovers mechanisms for growth instability

**DOI:** 10.1007/s10237-014-0569-5

**Published:** 2014-03-16

**Authors:** Ganarupan Satha, Stefan B. Lindström, Anders Klarbring

**Affiliations:** Mechanics, Department of Management and Engineering, The Institute of Technology, Linköping University, Linköping , 581 83 Sweden

**Keywords:** Goal function, Constrained mixture theory, Stability, Growth and remodeling, Blood vessel, Artery

## Abstract

A novel, goal function-based formulation for the growth dynamics of arteries is introduced and used for investigating the development of growth instability in blood vessels. Such instabilities would lead to abnormal growth of the vessel, reminiscent of an aneurysm. The blood vessel is modeled as a thin-walled cylindrical tube, and the constituents that form the vessel wall are assumed to deform together as a constrained mixture. The growth dynamics of the composite material of the vessel wall are described by an evolution equation, where the effective area of each constituent changes in the direction of steepest descent of a goal function. This goal function is formulated in such way that the constituents grow toward a target potential energy and a target composition. The convergence of the simulated response of the evolution equation toward a target homeostatic state is investigated for a range of isotropic and orthotropic material models. These simulations suggest that elastin-deficient vessels are more prone to growth instability. Increased stiffness of the vessel wall, on the other hand, gives a more stable growth process. Another important finding is that an increased rate of degradation of materials impairs growth stability.

## Introduction

The vascular system of the human body adapts to changing conditions, such as body mass changes, injury or disease. This is facilitated by the growth and remodeling of individual blood vessels in response to variations in blood pressure (Matsumoto and Hayashi 1996; Fridez et al. 2002; Hu et al. 2007a, b) and volumetric flow rate (Langille and O’Donnell 1986; Lehman et al. 1991; Langille et al. 1989; Brownlee and Langille 1991). While being intended to maintain an efficient and robust vascular system, some disease states lead to poorly controlled growth and remodeling, and consequently to the development of deformities in blood vessels. For example, abdominal aortic aneurysm (AAA) and thoracic and intracranial aneurysms are related to abnormal changes in the composition or morphology of vessels (Humphrey and Holzapfel 2012; Choke et al. 2005). In AAA, a part of the aorta becomes weakened due to thinning or changes in composition of the vessel wall that lead to a decrease in tensile strength, and ultimately to life-threatening rupture (Choke et al. 2005). The underlying mechanism that causes the development of growth instability is not fully elucidated, presumably due to the considerable complexity of the vessel growth control. Mathematical modeling of the growth control system offers a possibility for stability analyses, and for identifying the cause for instability and associated diseases. The present paper formulates such a mathematical model for dynamical processes of growth and remodeling of blood vessels, and investigates its stability for perturbations of a target homeostatic state. The model is based on constrained mixture theory (Humphrey and Rajagopal 2002; Gleason and Humphrey 2004; Valentín and Humphrey 2009a; Valentín et al. 2009), thin-walled tube theory, time-averaging over heartbeats, growth and remodeling evolution based on a goal function and a dynamical systems approach to optimization, all of which is described in more detail below.

Blood vessels consist of different constituents, such as elastin, collagen and smooth muscle (Boron and Boulpaep 2008, pp. 473–481). The mechanical properties of the blood vessel depend on the mechanical properties, the pretension and the amount of each constituent. Previously, the constrained mixture theory (CMT) has been employed to describe the composite material (Humphrey and Rajagopal 2002; Gleason and Humphrey 2004; Valentín and Humphrey 2009a; Valentín et al. 2009). In CMT, it is assumed that the constituents of the vessel deform together, so that they undergo the same incremental deformation.

Soft tissues in the body are produced and degraded continually. To account for this complication, the CMT has been extended to include the production and degradation of materials (Baek et al. 2006; Valentín et al. 2009). Added materials provide pretension, and their lifetime is described by a survival function. The individual constituents have different production and removal rates, which vary with the age of the person (Achakri et al. 1994; Stenmark and Mecham 1997). Moreover, the production and removal rates are actively controlled to enable the adaptation of vessels to a fluctuating environment.

The pressure difference across the vessel wall creates a circumferential stress in the vessel wall. A study on pigs shows that an experimentally controlled hypertension, that is an increase in the blood pressure, leads to an increased wall thickness of the basilar artery due to remodeling within two weeks (Hu et al. 2007b). A similar study on rats shows that hypertension modifies residual stresses (Matsumoto and Hayashi 1996). Cells in the vessel wall sense the stress and signal to the growth control system. The way in which the stress in the vessel wall affects growth has previously been modeled. In such models, it is assumed that there exists a target stress, called the homeostatic stress, and that growth occurs in such a way that this homeostatic stress is approached (Baek et al. 2005, 2006). One important input to the growth process is thus the difference between the current wall stress and the target wall stress (Baek et al. 2005, 2006).

Blood that flows through the vessel shears the endothelium of the vessel wall. The vessel senses the shear stress by means of mechanoreceptors on the surface of the endothelial cells (Ando and Yamamoto 2009; Johnson et al. 2011). Through a cascade of molecular signals emanating from the endothelium, the surrounding cells react and the vessel adapts its diameter to fit the level of blood flow (Lehoux et al. 2006; Ando and Yamamoto 2009; Johnson et al. 2011). Endothelial cells respond to changes in flow by releasing nitric oxide (NO), which makes the vessel dilate (vasodilatation) (Lehoux et al. 2006). Valentín and Humphrey (2009a, b) investigated the coupled roles of wall stress and shear stress by modeling how the difference between current and target values of these quantities influence the growth process of the vessel wall.

It is advantageous for the body’s vascular system to be able to rapidly adapt its blood vessel dimensions to varying conditions. This is facilitated by vascular smooth muscle cells (VSM), whose primary functions are to contract and dilate the blood vessel wall and thus to regulate the blood flow and pressure. There are limits to how much a blood vessel can expand or contract (Valentín et al. 2009; Valentín and Humphrey 2009a), and this means that large changes in vessel diameter can only be achieved by long-term growth and remodeling. VSM tension is controlled by biochemical substances: constrictors and dilators. The balance between these determines muscle tension, which was previously modeled (Valentín et al. 2009; Valentín and Humphrey 2009a, b). In that model, it is assumed that the vessel diameter is controlled by VSM so that the vessel instantaneously reaches its, in some sense, optimal diameter, if this can be achieved within the maximum dilation and contraction. The fractions of different vessel constituents then slowly adapt so that an optimal composition is reached.

Growth and remodeling of a biological system, such as the vascular system, are vastly complicated processes and mathematical models with predictive capability need to be formulated at a convenient level of complexity. As seen in classical models such as Wolff’s law of bone adaption (Frost 1994) and Murray’s law of dimensions of the vascular system (Murray 1926), a productive idea is to identify a global cost or objective function for the system. The optimal value of this function then gives what could be considered as a homeostatic state. In this paper, we are interested in evolutions of the system when perturbed away from such a homeostatic state. In similar work by Humphrey and co-workers (Baek et al. 2005, 2006; Valentín and Humphrey 2009b, a; Valentín et al. 2009), evolution equations are introduced by letting the mass production of different species depend on differences between actual and homeostatic stresses. Here, a somewhat different approach is employed: A goal function is assumed to govern also the evolution of the perturbed state. The evolution equation is then obtained by assuming that the state evolves along the direction of steepest descent. This approach is inspired by the so-called dynamical systems approach to optimization, which has previously been used for obtaining bone remodeling equations (Klarbring and Torstenfelt 2010; Klarbring and Torstenfel 2012a; Klarbring and Torstenfelt 2012b). For bone remodeling, the goal function is the total potential energy of the mechanical system, and we make a similar choice of goal function for soft tissues in the present paper.

We consider the blood vessel to be a thin-walled, cylindrical tube. For this model vessel, neither the geometry of the cross-section nor the composition of the vessel wall varies in the axial or the circumferential direction. We use CMT to model its mechanics and thus assume that the load-bearing constituents deform together, but have individual material behavior. The growth dynamics of the composite vessel wall material is described by the evolution of the effective cross-sectional areas of each constituent. The growth control is modeled using the dynamical systems approach to optimization. That is, the change in effective areas follows the direction of steepest descent of a goal function. We formulate this goal function in such way that the constituents grow toward a target potential energy and a target composition. The properties of the evolution equation are investigated numerically, using the parameters of the radial artery (*arteria radialis*), as obtained from in vivo and in vitro measurements found in the literature. We are interested in the stability of this dynamical system subject to changes in the flow conditions. Particularly, we consider under which conditions instabilities arise that cause the dimensions of the artery to drift in a manner similar to the development of an aneurysm.

## Theory

### Growth and remodeling control system

An important principle employed herein is that of locality of growth and remodeling: The evolution equation for growth and remodeling is formulated using variables of state that are local to a particular segment of artery. The state description comprises the composition and geometry of this vessel segment, collectively denoted by $\Omega$, and the local pressure $p$ and volumetric flow rate $u$, the latter being the volume of blood passing through a cross-section of the artery per unit time. Owing to this locality, the pressure and the flow rate are considered as given functions of time, since they are nonlocally controlled by the surrounding vascular system.

The remodeling process aims to ensure that a target geometry and composition, $\hat{\Omega }$, are approached and maintained. It is known from experiments (Matsumoto and Hayashi 1996; Fridez et al. 2002; Hu et al. 2007a, b; Langille and O’Donnell 1986; Lehman et al. 1991; Langille et al. 1989; Brownlee and Langille 1991) that the target blood vessel geometry and composition are strongly influenced by the flow conditions. Hypertension, for instance, leads to thickening and stiffening of the artery (Fung and Liu 1991; Matsumoto and Hayashi 1996). Other factors also regulate the target state, including longitudinal forces (Jackson et al. 2002). However, we limit the scope of this discussion to the pressure- and flow rate-dependence. The functional relation, $\hat{\Omega }(p,u)$, is tuned by evolution to optimize the probability for procreation, which is assumed to be achieved by minimizing the metabolic cost of the organism. In an early paper, Murray (1926) considered the cost of viscous losses of laminar blood flow and losses from maintaining the blood volume in a vessel. He showed that the cost-optimum infers a law relating the volumetric flow rate to the radius $r$ of the vessel: $r \propto u^{1/3}$. Subsequent work builds on this idea of cost-optimization in vascular systems (Klarbring et al. 2003; Kassab 2006; Lindström et al. 2014) and includes global optimization methods (Klarbring et al. 2003; Kassab 2006).

**Fig. 1:**
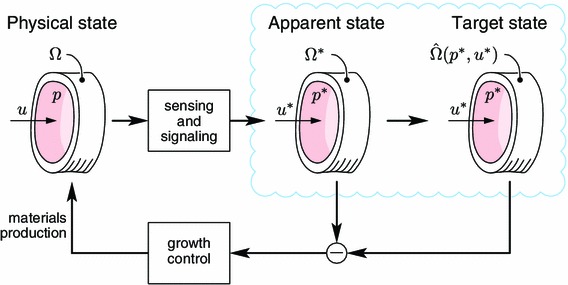
Control system for growth and remodeling. Sensing and signaling processes measure the physical state, $(\Omega ,p,u)$, which is perceived as an apparent state, $(\Omega ^*,p^*,u^*)$. The objective of growth control is to reduce the discrepancy between the apparent state and the target state, which is a function of the apparent flow conditions.

We proceed to schematically depict the growth control system at a high level of abstraction. This background enables us to specify the scope and limitations of our simplified model. The physical state of the vessel $(\Omega , p, u)$ is not directly available to the control system that regulates growth. Instead, sensing and signaling processes, including, e.g., mechanotransduction of epithelial cells (Paszkowiak and Dardik 2003), yield an apparent state $(\Omega ^*, p^*, u^*)$, which constitutes the vessel’s perception of its physical state, being the product of the signaling cascade. This apparent state could differ significantly from the physical state in case of, for instance, damage to the epithelial cells or the presence of plaque that interferes with the mechanotransduction. Growth is controlled by the discrepancy between the apparent $\Omega ^*$ and the target $\hat{\Omega }(p^*,u^*)$ composition and geometry. Note that the target $\hat{\Omega }$ is determined by the apparent flow conditions (Fig. 1). This distinction between the dynamics of sensing and signaling from the dynamics of growth enables separate stability analyses of those different systems, helping to identify different causes for disease.

As a first step toward a holistic model, the present investigation is limited to the function and stability of the growth control; the sensing and signaling dynamics are considered instantaneous and perfectly accurate. That is, the apparent and physical states are identical at all times: $(\Omega ^*, p^*, u^*)\equiv (\Omega , p, u)$.

### Constrained mixture mechanical model for a thin-walled tube

**Fig. 2:**
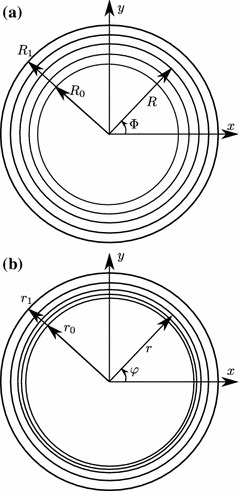
Geometry of the cross-section of a blood vessel in **a** the reference configuration and **b** the deformed configuration.

The mechanics of the vessel wall are an integral part of growth and remodeling; the transmural pressure and the vessel geometry are coupled through an equilibrium equation. Our model for growth and remodeling is based on a thin-walled tube assumption. However, we find it convenient and instructive to start our derivation from the basic thick-walled tube theory and then introduce the assumption leading to the thin-walled theory. The reference configuration of the tube is parameterized by cylindrical coordinates $(R,\varPhi ,Z)$ such that

1 $$\begin{aligned} R_0\le R\le R_1,\quad 0\le \varPhi \le 2\pi ,\quad 0\le Z\le L, \end{aligned}$$

where $R_0$ and $R_1$ are the inner and outer reference radii and $L$ is the reference length of the tube. This reference configuration is deformed so that a material point identified by $(R,\varPhi ,Z)$ is mapped to coordinates $(r,\varphi ,z)$ in the same cylindrical coordinate system (see Fig. 2):

2 $$\begin{aligned} r=r(R),\quad \varphi =\varPhi ,\quad z=\delta Z, \end{aligned}$$

for some function $r(R)$ and constant $\delta$. This means that the three principal axes of deformation are in the radial, circumferential and axial directions, and that the three principal stretches are

3 $$\begin{aligned} \lambda _r=\frac{\partial r}{\partial R},\quad \lambda =\lambda _{\varphi }=\frac{r}{R},\quad \lambda _z=\delta . \end{aligned}$$

We consider fluids to be an integral part of each constituent. That is, each constituent is itself a mixture of solids and fluid, at a fixed concentration. Owing to this presence of fluids, soft tissue is essentially volume-preserving. This is modeled using an incompressibility constraint $\lambda _r\lambda \lambda _z=1$, which gives

4 $$\begin{aligned} \lambda _r=(\lambda \lambda _z)^{-1}=\frac{R}{\delta r}. \end{aligned}$$

This volume conservation refers to the deformation behavior of existing materials. The addition or removal of materials through growth and remodeling is discussed in Sect. 2.4.

We use a constrained mixture theory, meaning that each constituent $k$ has the same deformation but different strain energy functions $\psi ^k$ which generally depend on the history of the material (Humphrey and Rajagopal 2002; Gleason and Humphrey 2004; Valentín and Humphrey 2009a; Valentín et al. 2009). Moreover, each constituent is considered to be an incompressible material that is orthotropic with respect to axial and circumferential directions, meaning that the strain energy of each constituent can be considered as functions of the two stretches $\lambda$ and $\lambda _z$, and that the constitutive equations read, cf. Ogden (Holzapfel and Ogden 2003),

5a $$\begin{aligned} \sigma _\varphi ^k-\sigma _r^k&= \lambda \frac{\partial \psi ^k}{\partial \lambda },\end{aligned}$$

5b $$\begin{aligned} \sigma _z^k-\sigma _r^k&= \lambda _z\frac{\partial \psi ^k}{\partial \lambda _z}, \end{aligned}$$

for the individual constituent principal stresses $\sigma _r^k, \sigma _\varphi ^k$ and $\sigma _z^k$. According to the mixing rule, the total stresses are defined as:

6 $$\begin{aligned} \sigma _\varphi =\sum _k\phi ^k\sigma _\varphi ^k,\quad \sigma _r=\sum _k\phi ^k\sigma _r^k,\quad \sigma _z=\sum _k\phi ^k\sigma _z^k, \end{aligned}$$

where $\phi ^k$ is the volume fraction of constituent $k$.

The equilibrium equation for the radial direction in a cylindrical coordinate system reads

7 $$\begin{aligned} \frac{\partial \sigma _r}{\partial r}+\frac{\sigma _r-\sigma _\varphi }{r}=0. \end{aligned}$$

Integrating and taking into account the boundary conditions $\sigma _r=-p$ at $r_0=r(R_0)$ and $\sigma _r=0$ at $r_1=r(R_1)$, we obtain

8 $$\begin{aligned} p=\int \limits _{r_0}^{r_1}(\sigma _\varphi -\sigma _r)\frac{\mathrm{d}r}{r}, \end{aligned}$$

where $p$ is the time-dependent pressure difference between the interior and the exterior. Introducing the constitutive Eq. (5a), we obtain

9 $$\begin{aligned} p=\int \limits _{r_0}^{r_1}\lambda \left( \frac{\partial }{\partial \lambda }\sum _k\phi ^k\psi ^k\right) \frac{ \mathrm{d}r}{r}. \end{aligned}$$

We rewrite the integral in Eq. (9) in terms of the reference coordinate $R$ by using $r=\lambda R$ and $dr=(\lambda \lambda _z)^{-1}dR$, and introduce the assumption, approximately valid for thin-walled tubes, that

10 $$\begin{aligned} (\lambda \lambda _z)^{-1}\frac{\partial }{\partial \lambda }\sum _k\phi ^k\psi ^k \end{aligned}$$

is constant through the thickness. Equation (9) then becomes

11 $$\begin{aligned} p=\frac{1}{2\pi \lambda \lambda _zR_0^2}\frac{\partial }{\partial \lambda }\sum _kA^k\psi ^k, \end{aligned}$$

where

12 $$\begin{aligned} A^k=2\pi \phi ^kR_0^2\ln \frac{R_1}{R_0} \end{aligned}$$

is essentially the effective area of constituent $k$. In fact, for $H\equiv R_1-R_0\ll R_0$, it holds that

13 $$\begin{aligned} A^k\approx 2\pi \phi ^kR_0H\approx \pi \phi ^k(R_1^2-R_0^2). \end{aligned}$$

For a given pressure and material composition, Eq. (11) contains two unknown deformations. However, an assumption that is reasonable in many applications is that $\delta =\lambda _z$ is constant. From here on, we assume that the strain energy is a function of $\lambda$ only. With the definitions and assumptions introduced above, the geometry and composition of a blood vessel are described by $\Omega = (\lambda , \mathbf {A})$, where $\mathbf {A}$ is the vector of effective areas.

### Time-averaging

Growth and remodeling are low-frequency processes as compared to the heartbeat. We define the time window-average of a quantity $y$ by

14 $$\begin{aligned} \langle y \rangle = \frac{1}{T} \int \limits _{t-T/2}^{t+T/2} y(\tau ) d \tau . \end{aligned}$$

Generally, $\langle y \rangle$ depends on both the time window $T$ and the time $t$. If the function $y$ is a superposition of a fast process, with characteristic timescale much less than $T$, and a slow process, with characteristic timescale much greater than $T$, we may write $y = \langle y \rangle + \tilde{y}$, where $\tilde{y}$ represents the fluctuations of the high-frequency process (Appendix 1).

For the growth of blood vessels, we take $T$ to be much greater than the period of the heartbeat, yet much smaller than the typical timescales of growth and remodeling. It is assumed that $A^{k}\approx \langle A^{k} \rangle$, since the remodeling process is slow. By contrast, the volumetric flow rate $u=\langle u\rangle +\tilde{u}$, pressure $p=\langle p \rangle +\tilde{p}$ and stretch $\lambda = \langle \lambda \rangle +\tilde{\lambda }$ are rapidly varying quantities. Consider the equilibrium Eq. (11). It may be written

15 $$\begin{aligned} p = \Upsilon \circ \lambda ,\quad \Upsilon (\lambda )=\frac{1}{2\pi \lambda _{z}R^{2}_{0}}\frac{1}{\lambda }\frac{\partial }{\partial \lambda }\sum _kA^k\psi ^k(\lambda ), \end{aligned}$$

where ‘$\circ$’ denotes function composition. Taking the time window-average of Eq. (15) gives

16 $$\begin{aligned} \langle p \rangle = \langle \Upsilon \circ \lambda \rangle \approx \big \{\text {Eq.}(53) \big \} \approx \Upsilon \circ \langle \lambda \rangle , \end{aligned}$$

so that a first-order approximation reads

17 $$\begin{aligned} \langle p \rangle =\frac{1}{2\pi \lambda _{z}R^{2}_{0} \langle \lambda \rangle } \frac{\partial }{\partial \langle \lambda \rangle }\sum _{k}A^{k}\psi ^k(\langle \lambda \rangle ). \end{aligned}$$

Thus, the equilibrium equation is approximately valid for the time-average pressure and stretch. Errors originate from nonlinearities in $\Upsilon$, as evident from the structure of the error terms of Eq. (52) in Appendix 1. Henceforth, we omit the averaging brackets, understanding that the ensuing discussion concerns time-average quantities.

### The evolution of effective areas and strain energy functions

In this section, we are interested in the growth dynamics of the multicomponent material of a blood vessel. The evolution of the effective areas comprises two terms: The remainder of the original materials and the remainder of the materials $\fancyscript{A}^{k}d\tau$ formed at time $\tau$. Baek et al. (2006) formulated these growth dynamics as an evolution of mass per unit reference area. An equivalent formulation using effective areas is obtained by dividing the mass per unit reference area of each species by its constant density, and then multiplying this obtained thickness by a referential circumference, giving

18 $$\begin{aligned} A^{k}=A^{k}(0)Q^{k}(t)+\int \limits ^{t}_{0}\fancyscript{A}^{k}(\tau )q^{k}(t-\tau )d\tau ,\quad t \ge 0 \end{aligned}$$

where $A^{k}(0)$ is the original effective area of constituent $k, Q^{k}(t)$ is the fraction of constituent $k$ that was produced before time $0$ and remains at time $t, \fancyscript{A}^{k}(t)\ge 0$ is the rate of production of effective area at time $t$ and $q^{k}(t)$ is a monotonically decreasing survival function such that $q(0)=1$. Differentiation of Eq. (18) with respect to $t$, gives

19 $$\begin{aligned} \frac{\mathrm{d}A^{k}}{\mathrm{d}t}=\fancyscript{A}^{k}+\fancyscript{A}^{k}_\mathrm {d}, \end{aligned}$$

where

20 $$\begin{aligned} \fancyscript{A}^{k}_\mathrm {d} = A^{k}(0)\mathrm{d}Q^{k}(t) + \int \limits ^{t}_{0} \fancyscript{A}^{k}(\tau )dq^{k}(t-\tau )d\tau , \end{aligned}$$

represents the rate of change of effective area due to degradation of constituent $k$. Here, and in the following, we use the notation $\mathrm{d}f(s)=\mathrm{d}f/\mathrm{d}s$.

By assuming that materials created at different time instances contribute to the strain energy in proportion to remaining area fractions, we obtain (Baek et al. 2006)

21 $$\begin{aligned} A^{k}\psi ^{k}(\lambda )&= A^{k}(0)Q^{k}(t)\Psi ^{k}\left[ \lambda ^{k}(t,0)\right] \nonumber \\&+\quad \int \limits ^{t}_{0}\fancyscript{A}^{k}(\tau )q^{k}(t-\tau )\Psi ^{k}\left[ \lambda ^{k}(t,\tau )\right] d\tau , \end{aligned}$$

where $\Psi ^{k}\left[ \lambda ^{k}(t,\tau )\right]$ is the strain energy density with respect to a natural, stress-free configuration and characterizes the nonlinear, elastic behavior (Baek et al. 2006). Also, $\lambda ^{k}(t,\tau )$ is the stretch at time $t$ for materials produced at time $\tau$, i.e., (Baek et al. 2006)

22 $$\begin{aligned} \lambda ^{k}(t,\tau )=\frac{\lambda (t)}{\lambda (\tau )}G^k_\mathrm {h}. \end{aligned}$$

The ratio $\lambda (t)/\lambda (\tau )$ is the stretch developed during growth and $G^k_\mathrm {h}$ is the homeostatic prestretch of constituent $k$, i.e., the material may attain prestretch at its time of production.

### Homeostatic conditions

When the imposed flow conditions $u$ and $p$ are unchanging, the time-average composition and geometry of the vessel may evolve into a steady state. We identify this as the homeostatic state. Also, when the flow conditions are varying much slower than the timescales of remodeling, the vessel is essentially in a homeostatic state at each point in time. The homeostatic stretch is taken to be a constant $\lambda =\tilde{\lambda }$. The target state $\hat{\Omega } = (\hat{\lambda }, \hat{\mathbf {A}})$ is such a homeostatic state. There are two classes of constituents for which steady-state conditions are possible:

i. Constituents that degrade, $Q^k,q^k \rightarrow 0$ as $t\rightarrow \infty$, and grow, $\fancyscript{A}^k \ge 0$.
ii. Constituents that neither degrade, $Q^k = 1$, nor grow, $\fancyscript{A}^k = 0$.

The set of constituent indices belonging to class (i) and (ii) are denoted by $S_\mathrm {i}$ and $S_\mathrm {ii}$, respectively. In case (i), Eq. (18) reduces to

23 $$\begin{aligned} A^{k}=\int \limits ^{\infty }_{0}\fancyscript{A}^{k}(\tau )q^{k}(t-\tau )d\tau , \end{aligned}$$

and Eq. (21) becomes

24 $$\begin{aligned} A^{k}\psi ^{k}(\lambda )=\Psi ^{k}\left( \frac{\lambda }{\tilde{\lambda }}G^k_\mathrm {h}\right) \int \limits ^{\infty }_{0}\fancyscript{A}^{k}(\tau )q^{k}(t-\tau )d\tau . \end{aligned}$$

Inserting Eq. (23) into Eq. (24) gives

25 $$\begin{aligned} \psi ^{k}(\lambda )=\Psi ^k\left( \frac{\lambda }{\tilde{\lambda }}G^k_\mathrm {h}\right) , \quad k \in S_\mathrm {i}. \end{aligned}$$

In case (ii), Eq. (18) yields $A^{k}=A^{k}(0)$, which is inserted into Eq. (21), becoming

26 $$\begin{aligned} A^{k}\psi ^{k}(\lambda )=A^{k}Q^{k}(t)\Psi ^{k}\left[ \frac{\lambda }{\lambda (0)}G^k_\mathrm {h}\right] \quad \Leftrightarrow \nonumber \\ \psi ^{k}(\lambda )= \Psi ^{k}\left[ \frac{\lambda }{\lambda (0)}G^k_\mathrm {h}\right] . \end{aligned}$$

Since $\lambda$ is the stretch with respect to the reference configuration, $G_{\mathrm {h}}^k/\lambda (0)$ is the stretch given by deforming the natural stress-free configuration of constituent $k\in S_{\mathrm {ii}}$ into the reference configuration. This may be seen as a definition of $G_{\mathrm {h}}^k$ in case of $k\in S_{\mathrm {ii}}$. In order to treat both cases (i) and (ii) by the same notation, we choose to define the prestretch for nongrowing constituents so that $\lambda (0) = \tilde{\lambda }$, yielding

27 $$\begin{aligned} \psi ^{k}(\lambda )=\Psi ^k\left( \frac{\lambda }{\tilde{\lambda }} G_{\mathrm {h}}^k \right) , \quad k \in S_\mathrm {ii}. \end{aligned}$$

Note that, in both cases (i) and (ii), we then have $\lambda ^k = G_{\mathrm {h}}^k$ in the homeostatic state according to Eq. (22).

Introducing Eq. (25) or Eq. (27) into the time-averaged equilibrium Eq. (17), and evaluating for $\lambda =\lambda (t)=\lambda (\tau )=\tilde{\lambda }$, we get

28 $$\begin{aligned} p=\frac{1}{2\pi \lambda _z(R_0\lambda )^2}\sum _kA^k\sigma _\mathrm {h}^{k}(\lambda ), \end{aligned}$$

where

29 $$\begin{aligned} \sigma _\mathrm {h}^{k}(\lambda ) = G^k_\mathrm {h}d{\Psi }^k(G^k_\mathrm {h}) \end{aligned}$$

is called the homeostatic stress.

### Growth toward a target state

As described in Sect. 2.1, we now assume that the flow conditions, $p(t)$ and $u(t)$, are given functions and consider how the target state is approached and maintained by the growth process.

As indicated in the introduction, we approach the formulation of equations for growth in a novel way, assuming the existence of a goal function and an evolution in a steepest descent direction of this function. As a measure of the mechanical state, we then use the total potential energy of the system, which reads

30 $$\begin{aligned} \Pi \left( \lambda ,\mathbf {A} \right) =\sum _{k} A^{k}\psi ^{k} - \pi R^{2}_{0}\lambda _{z}\lambda ^{2} p. \end{aligned}$$

It is straight-forward to verify that $\Pi$ has a stationary value with respect to $\lambda$, that is $\partial \Pi /\partial \lambda =0$ by virtue of the equilibrium Eq. (17) for time-average quantities. As an objective or goal function, we will now use

31 $$\begin{aligned} f(\mathbf {A})=\frac{1}{2}\left[ \Pi (\lambda ,\mathbf {A}) - \hat{\Pi }\right] ^{2}+\frac{1}{2} \sum _{k} b^k\left[ A^{k}-\hat{A}^{k}\right] ^{2}, \end{aligned}$$

where $\lambda =\lambda (\mathbf {A})$ from Eq. (17), $b^k > 0$ are constants with units $[\mathrm{kg}^2 \mathrm{m}^{-2}\mathrm{s}^{-4}]$ and $\hat{\Pi }=\Pi (\hat{\lambda },\hat{\mathbf {A}})$. This proposed goal function is based on a prior knowledge of the target state $\hat{\Omega }=(\hat{\lambda },\hat{\mathbf {A}})$. This target state can be seen as defined by a separate optimization process where, in the spirit of Murray (1926), the work associated with pumping and maintaining blood, as well as maintaining the material of the artery wall, is included. This optimization problem is formulated and solved in a separate paper (Lindström et al. 2014). In the present paper, we study perturbations away from the target state, and it is therefore natural to use the least-squares type goal function of Eq. (31). However, $\mathbf {A}$ and $\lambda$ are coupled through the equilibrium Eq. (17), which will be a constraint in the optimization process. An effective way of handling equilibrium constraints in optimization has been developed in structural optimization (Christensen and Klarbring 2009): The potential energy is used as goal function and, since this function has a stationary value at equilibrium, the constraint (17) is implicitly satisfied. We develop here a similar method for satisfying equilibrium by using a target potential energy instead of the more direct target strain $\hat{\lambda }$.

The partial derivatives of the goal function are calculated as follows:

32 $$\begin{aligned} \frac{\partial f}{\partial A^{k}}&= \left[ \Pi (\lambda ,\mathbf {A})-\hat{\Pi }\right] \left[ \frac{\partial \Pi }{\partial \lambda }\frac{\partial \lambda }{\partial A^{k}}+\frac{\partial \Pi }{\partial A^{k}}\right] \nonumber \\&+\, b^k\left[ A^{k}-\hat{A}^{k}\right] \nonumber \\&= \left[ \Pi (\lambda ,\mathbf {A})-\hat{\Pi }\right] \psi ^{k} + b^k\left[ A^{k}-\hat{A}^{k}\right] , \end{aligned}$$

where it becomes clear that the property $\partial \Pi /\partial \lambda =0$ facilitates the computation while it makes ${\partial \lambda }/{\partial A^{k}}$, which would otherwise follow from the implicit derivation of the equilibrium equation, disappear. This is another reason for choosing this particular form of the goal function.

It is now assumed that the gradient of the goal function governs the evolution toward a steady state. The simplest assumption that achieves this is given by

33 $$\begin{aligned} \frac{d A^k}{dt}=\left\{ \begin{array}{ll} -C\frac{\partial f}{\partial A^k}, &{}\quad \text {if}\quad -C\frac{\partial f}{\partial A^k} \ge \fancyscript{A}^{k}_\mathrm{d} \\ \fancyscript{A}^{k}_\mathrm{d}, &{}\quad \text {otherwise}. \end{array}\right. \end{aligned}$$

where $C$ is a positive constant with units $[\mathrm{m}^2\mathrm{s}^3\mathrm{kg}^{-2}]$. In Eq. (33), the loss of materials is limited by the rate of degradation [see Eq. (20)]. This enforces the condition $\fancyscript{A}^{k} \ge 0$ on the rate of production. It is also conceivable to use a general function of the areas instead of the constant $C$. Crucially, elastin degrades very slowly and is not produced in adult individuals (Tsamis et al. 2013). For such essentially static constituents, we set $\mathrm{d} A^k / \mathrm{d}t = 0$. That is, the growth and remodeling process does not attempt to control static effective areas.

## Numerical experiments

The properties of the evolution equation are investigated numerically, using the parameters of the radial artery (*arteria radialis*). Averaging in vivo measurements yields a radius $r_0=1.265$ mm and transmural pressure $p_0 = 12$ kPa for the radial artery (Laurent et al. 1994). Moreover, the average circumferential stress is $\sigma _0 = 52.6$ kPa and that the incremental modulus of the vessel wall is $E_0 = 2.68$ MPa at this average point of operation (Laurent et al. 1994).

### Material models

The deformations of the materials of the vessel wall are assumed to be volume-preserving (Sect. 2.2). To fully describe the material properties of the constituents, we have left to specify their strain energy functions. As previously described (Holzapfel et al. 2000; Holzapfel and Ogden 2010), the stretches of the constituents are $\lambda ^k, (\lambda ^k\lambda _z)^{-1}$ and $\lambda _z$, so that the Cauchy–Green tensor is

34 $$\begin{aligned} \mathbf {C}^k = \left[ \begin{array}{ccc} (\lambda ^k)^2 &{} 0 &{} 0 \\ 0 &{} (\lambda ^k\lambda _z)^{-2} &{} 0 \\ 0 &{} 0 &{} \lambda _z^2 \\ \end{array} \right] . \end{aligned}$$

We choose an anisotropic, nonlinear elastic material model for the constituents of the vessel wall, as previously proposed (Holzapfel and Ogden 2010). A variant of this model includes two preferred collagen fibril directions (Pandolfi and Holzapfel 2008), as found in, e.g., the aorta (Schriefl et al. 2012), but here we take the preferred fibril direction to be circumferential, for simplicity. Each constituent $k$ is then modeled by the strain energy function (Holzapfel and Ogden 2010)

35 $$\begin{aligned} \Psi ^k(I^k_0,I^k_1) = \Psi ^k_{\mathrm {g}}(I^k_0) + \Psi ^k_{\mathrm {f}}(I^k_1), \end{aligned}$$

where $I^k_0 = \mathrm {tr}\,\mathbf {C}^k$ and $I^k_1 = (\lambda ^k)^2$. The two terms of the strain energy represent an isotropic component

36 $$\begin{aligned} \Psi ^k_{\mathrm {g}}(I^k_0) = \frac{c^k_0}{2}(I^k_0 - 3), \end{aligned}$$

and an anisotropic component that only depends on the stretch in the circumferential direction

37 $$\begin{aligned} \Psi ^k_{\mathrm {f}}(I^k_1) = \frac{c^k_1}{2 c^k_2} \left\{ \exp \left[ c^k_2 (I^k_1 - 1)^2 \right] - 1 \right\} , \end{aligned}$$

where $c^k_0 \ge 0$ and $c^k_1 \ge 0$ are constants with the units of stress, and $c^k_2 > 0$ is a nondimensional constant. Each constituent $k$ is thus associated with three material parameters.

### Parameter identification

The mechanical properties of the vessel wall are characterized by the incremental modulus $E_{\mathrm {inc}} = d\sigma _{\mathrm {w}}/d\varepsilon _{\mathrm {w}}$ measured by Laurent et al. (1994), where $\sigma _{\mathrm {w}}$ is the circumferential wall stress and $\varepsilon _{\mathrm {w}}$ is the circumferential wall strain with respect to a vessel with zero transmural pressure. To fit the material model parameters, there is a need to derive the expressions $\sigma _{\mathrm {w}}(\varepsilon _{\mathrm {w}})$ and $E_{\mathrm {inc}}(\varepsilon _{\mathrm {w}})$ within our modeling frame.

The wall stress $\sigma _{\mathrm {w}}$ is defined as pressure times the ratio between vessel radius $\lambda R_0$ and wall thickness $\sum _k A^k / 2 \pi \lambda R_0$. The equilibrium Eq. (17) then gives

38 $$\begin{aligned} \sigma _{\mathrm {w}} = \frac{2 \pi \lambda ^2 R_0^2 p}{\sum _k A^k} = \frac{\lambda }{\lambda _z} \sum _k \phi ^k \frac{\partial \psi ^k}{\partial \lambda }. \end{aligned}$$

The measured vessel is assumed to be in a homeostatic state. For this in vivo homeostatic state, $\phi ^k$ are constants and the unchanging time-average strain $\lambda =\tilde{\lambda }$ implicitly defines a reference geometry. Moreover, we have (Sect. 2.5)

39 $$\begin{aligned} \psi ^k(\lambda ) = \Psi ^k\left( \frac{\lambda }{\tilde{\lambda }} G_{\mathrm {h}}^k \right) , \end{aligned}$$

so that the wall stress of Eq. (38) is written

40 $$\begin{aligned} \sigma _{\mathrm {w}}\left( \frac{\lambda }{\tilde{\lambda }}\right) = \frac{1}{\lambda _z} \frac{\lambda }{\tilde{\lambda }} \sum _k \phi ^k G_{\mathrm {h}}^k d\Psi ^k\left( \frac{\lambda }{\tilde{\lambda }} G_{\mathrm {h}}^k \right) . \end{aligned}$$

The wall strain $\varepsilon _{\mathrm {w}}$ with respect to the unloaded state of the extracted vessel is defined so that it is linear in $\lambda$ and so that $\varepsilon _{\mathrm {w}} = 0$ when $\sigma _{\mathrm {w}}=0$. This gives

41 $$\begin{aligned} \varepsilon _{\mathrm {w}} = \frac{\lambda }{\tilde{\lambda }\lambda _{\mathrm {eq}}} - 1, \end{aligned}$$

where $\lambda _{\mathrm {eq}}$ is implicitly defined by the Eq. $\sigma _{\mathrm {w}}(\lambda /\tilde{\lambda } = \lambda _{\mathrm {eq}}) = 0$ for the unstressed material. Inserting $\lambda /\tilde{\lambda } = \lambda _{\mathrm {eq}}(1+\varepsilon _{\mathrm {w}})$ into Eq. (40) gives

42 $$\begin{aligned} \sigma _{\mathrm {w}}(\varepsilon _{\mathrm {w}}) = \frac{\lambda _{\mathrm {eq}}}{\lambda _z} (1 + \varepsilon _{\mathrm {w}}) \sum _k \phi ^k G_{\mathrm {h}}^k d\Psi ^k\left[ \lambda _{\mathrm {eq}} G_{\mathrm {h}}^k(1 + \varepsilon _{\mathrm {w}}) \right] . \end{aligned}$$

The incremental modulus is then computed from its definition,

43 $$\begin{aligned} E_{\mathrm {inc}}(\varepsilon _{\mathrm {w}})&= \frac{\sigma _{\mathrm {w}}(\varepsilon _{\mathrm {w}})}{1+\varepsilon _{\mathrm {w}}} + \frac{\lambda _{\mathrm {eq}}^2(1 + \varepsilon _{\mathrm {w}})}{\lambda _z} \nonumber \\&\quad \sum _k \phi ^k (G_{\mathrm {h}}^k)^2 d^2\Psi ^k\left[ \lambda _{\mathrm {eq}}G_{\mathrm {h}}^k(1 + \varepsilon _{\mathrm {w}}) \right] . \end{aligned}$$

The material models we use must be as simple as possible to yield generic insights into the dynamics of growth and remodeling, while still capturing the salient features of the mechanics. We investigate three different material models below: A one-constituent, isotropic material model; a one-constituent, orthotropic material model; and a two-constituent model with one isotropic material that does not grow or degrade (elastin), and one anisotropic material that degrades and grows.

In each case, there is only one constituent, $k=1$, that grows and remodels. For this constituent, we take the survival functions to be

44 $$\begin{aligned} Q^1(t) = q^1(t) = e^{-\nu ^1 t}, \end{aligned}$$

where $\nu ^1$ is a rate constant capturing the turnover of this constituent. The body uses enzymes to regulate the rate of degradation (Campbell et al. 1999 pp. 91–94). However, we assume that $\nu ^1$ is unchanging in space and time, for simplicity. We choose $\nu ^1=\ln 2 / 60\,\mathrm day ^{-1}$, consistent with the half-life of collagen (Martufi and Gasser 2012; Nissen et al. 1978). We also assume that $\lambda _z = 1$ in each case.

#### One-constituent isotropic model

While unrealistic, investigating the simple case of one constituent is useful for understanding the basic properties of the evolution equation. It is thus assumed that the vessel consists of a single, isotropic material component. We use Eq. (35), only retaining the isotropic term, i.e., $c_1^k = 0$. The expression for the potential strain energy becomes

45 $$\begin{aligned} \Psi ^1(\lambda ^1) = \frac{c_0^1}{2}\left[ (\lambda ^1)^2 + (\lambda ^1)^{-2} - 2 \right] . \end{aligned}$$

The homeostatic prestretch of this model material is set as the homeostatic prestretch of collagen, $G_{\mathrm {h}}^1=1.08$ (Valentín and Humphrey 2009a, b; Valentín et al. 2009). For this one-constituent case, we solve Eq. (40) for $\lambda _{\mathrm {eq}}=\lambda /\tilde{\lambda }$ when $\sigma _{\mathrm {w}}=0$. That is, we solve

46 $$\begin{aligned} d\Psi ^1\left( \lambda _{\mathrm {eq}} G_{\mathrm {h}}^1 \right) = 0, \end{aligned}$$

and because $\Psi ^1(\lambda ^1)$ has a stationary point at $\lambda ^1 = 1$, we obtain $\lambda _{\mathrm {eq}}=1/G_{\mathrm {h}}^1$. This is inserted into Eq. (41), giving $\varepsilon _{\mathrm {w}}=G_{\mathrm {h}}^1\lambda /\tilde{\lambda }-1$ for the one-constituent material. We assume that we have a homeostatic state, $\lambda /\tilde{\lambda } = 1$, at the average point of operation. Therefore, we can compute the parameter $c_0^1$ from the Eq. $E_{\mathrm {inc}}(\varepsilon _{\mathrm {w}}=G_{\mathrm {h}}^1-1;c_0^1) = E_0$, thus equating the incremental modulus with its experimentally observed value at this average point of operation.

#### One-constituent orthotropic material

**Fig. 3:**
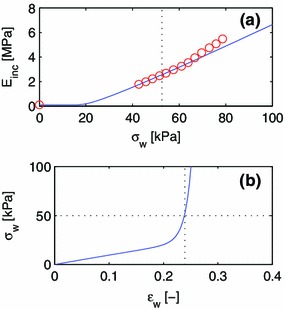
Least-squares fit of a one-constituent, orthotropic material model to experimental data by Laurent et al. (1994) $(\sigma _{\mathrm {w}} > 0)$ and Girerd et al. (1998) $(\sigma _{\mathrm {w}} = 0)$. The data point at $\sigma _{\mathrm {w}}=0$ as well as the eight data points closest to the point of operation (*dashed line*) is used for the fit.

In a slightly improved model, we view the wall of the radial artery as one anisotropic material $k=1$, to establish the material properties of this composite. The material then has the potential energy of Eq. (35) with three nonzero parameters $c_0^1, c_1^1$ and $c_2^1$. The parameterized curve defined by Eqs. (42) and (43) is least-squares fitted to measurements of $E_{\mathrm {inc}}(\sigma _{\mathrm {w}})$ by Laurent et al. (1994), as shown in Fig. 3. Laurent et al. (1994) used an ultrasound device to measure the lumen diameter and the wall thickness as functions of time. Simultaneous, noninvasive measurements of finger blood pressure made it possible to compute $E_{\mathrm {inc}}$ as a function of circumferential wall stress. The measurements that we use are ensemble averages for 22 normotensive subjects between the ages of 25 and 64 in a supine position after 20 minutes’ rest. The parameter $c_0$ is not very sensitive to the data points in the range of strains of the operating window of the artery. Therefore, we also use an in vitro measurement for $E_{\mathrm {inc}}(\varepsilon _{\mathrm {w}} = 0) = 0.10$ MPa (Girerd et al. 1998) to eliminate $c_0$ as a free parameter by enforcing the relation $E_{\mathrm {inc}}(0) = 4(c^1_0 + c_1^1)$ derived from the material model. Girerd et al. (1998) measured the incremental modulus of surgically removed sections of the radial artery, using essentially the same measurement technique as Laurent et al. (1994) but with an experimentally controlled transmural pressure. The material parameters from the fit are compiled in Table 1, along with the homeostatic prestretch obtained by finding the stretch at which the wall stress of the model material equals the average wall stress, $\sigma _0=52.6$ kPa (Laurent et al. 1994), of the radial artery.

**Table 1:** Material properties of constituents of blood vessels

Material	*k*	$c_0^k$	$c_1^k$	$c_2^k$	$G_{\mathrm {h}}^k$
		[kPa]	[Pa]	[$-$]	[$-$]
1-constituent, isotropic	1	715	$-$	$-$	$1.08^{a}$
1-constituent, orthotropic	1	25.0	5.74	28.1	1.24
2-constituent, anisotropic	1	$-$	50.5	50.7	1.167
2-constituent, isotropic	2	88.8	$-$	$-$	$1.40^{b}$

The two entries of the two-constituent model represent different components of the composite material

$^{a}$Valentín and Humphrey (2009a, b); Valentín et al. (2009)

$^{b}$Valentín and Humphrey (2009a, b); Valentín et al. (2009)

#### Two-constituent model

It is assumed that the vessel wall can be divided into two components: One component, $k=1$, represents the materials that primarily contribute to the anisotropic term of the strain energy, with $c_0^1 = 0$. This component includes smooth muscle and collagen. The second component, $k=2$, represents the materials of the isotropic term of the strain energy, with $c_1^2 = 0$; this is essentially elastin. Histological data for the radial artery from the literature (Li et al. 2008) are used to estimate the respective fractions of the isotropic and anisotropic constituents. The fraction of elastin (isotropic fraction) is $\phi ^2=0.1627$, while the fraction of smooth muscle and collagen (anisotropic fraction) is $\phi ^1=0.8373$. The effects of other materials are neglected. The two-component model material is then described by three parameters: $c_1^1$ and $c_2^1$ of the anisotropic fraction, and $c_0^2$ of the isotropic fraction.

**Fig. 4:**
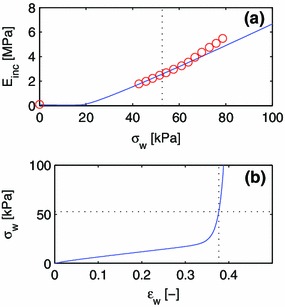
Least-squares fit of a two-constituent material model to experimental data by Laurent et al. (1994) $(\sigma _{\mathrm {w}} > 0)$ and Girerd et al. (1998) $(\sigma _{\mathrm {w}} = 0)$. The data point at $\sigma _{\mathrm {w}}=0$ as well as the eight data points closest to the point of operation (*dashed line*) is used for the fit.

Again, it is required that $E_{\mathrm {inc}}(\varepsilon _{\mathrm {w}} = 0; c_0^2,c_1^1, c_2^1) = 0.10$ MPa (Girerd et al. 1998). Particularly, the parameterized curve defined by Eqs. (42) and (43) is least-squares fitted in $(c_1^1, c_2^1)$-space to experimental data (Laurent et al. 1994), and the equation $E_{\mathrm {inc}}(\varepsilon _{\mathrm {w}} = 0; c_0^2,c_1^1, c_2^1) = 0.10$ MPa is solved for $c_0^2$ for each given pair $(c_1^1, c_2^1)$ explored in the fitting procedure.The isotropic component is assumed to mainly comprise elastin, whose prestretch is $G_{\mathrm {h}}^2 = 1.40\,$(Valentín and Humphrey 2009b, a; Valentín et al. 2009). The prestretch of the composite anisotropic constituent is unknown. Therefore, fits were conducted for different values of $G_{\mathrm {h}}^1$ and the bisection method was employed to find the value, $G_{\mathrm {h}}^1=1.167$, at which the homeostatic wall stress becomes $\sigma _{\mathrm {w}} = \sigma _0 = 52.6$ kPa (Laurent et al. 1994). See Fig. 4 for the resulting fit.

### Simulations

#### Nondimensional evolution equation

To identify the parameters of the dynamical system, its evolution equation is nondimensionalized by introducing a referential length $L_{\mathrm {ref}} = R_0$, a referential time $t_{\mathrm {ref}} = (\nu ^1)^{-1}$ and a referential mass $m_{\mathrm {ref}} = R_0 E_0 (\nu ^1)^{-2}$, where $E_0$ denotes the average incremental elastic modulus observed in experiments for the radial artery at the point of operation. Here, the referential mass was chosen so that the derived referential stress becomes $m_{\mathrm {ref}}/L_{\mathrm {ref}}t_{\mathrm {ref}}^{2} = E_0$.

An index ‘$\times$’ is used to indicate nondimensional quantities, so that

47a $$\begin{aligned} t_{\times }&= \frac{t}{t_{\mathrm {ref}}} = \nu ^1 t,\end{aligned}$$

47b $$\begin{aligned} A^k_{\times }&= \frac{A^k}{L_{\mathrm {ref}}^2} = \frac{A^k}{R_0^2},\end{aligned}$$

47c $$\begin{aligned} f_\times&= \frac{f t_{\mathrm {ref}}^4}{m_{\mathrm {ref}}^2 L_{\mathrm {ref}}^2} = \frac{f}{E_0^2 R_0^4}, \end{aligned}$$

where the goal function has the units of force squared. The evolution Eq. (33) for constituent $k=1$ is then written

$$\begin{aligned} \frac{\mathrm{d} A^1}{\mathrm{d}t}=-C\frac{\partial f}{\partial A^1} \quad \Leftrightarrow \quad \frac{\mathrm{d} (A^1_\times R_0^2)}{\mathrm{d}(t_\times /\nu ^1)}=-C\frac{\partial (f_\times E_0^2 R_0^4)}{\partial (A^1_\times R_0^2)} \quad \Leftrightarrow \end{aligned}$$

48 $$\begin{aligned} \frac{\mathrm{d} A^1_\times }{\mathrm{d}t_\times }=- \frac{CE_0^2}{\nu ^1} \frac{\mathrm{d} f_\times }{\mathrm{d} A^1_\times } = - \frac{CE_0^2}{\nu ^1} \frac{\Pi - \hat{\Pi }}{E_0 R_0^2} \frac{\psi ^{1}}{E_0} - \frac{b^1C}{\nu ^1}\frac{A^{1}-\hat{A}^{1}}{R_0^2}, \end{aligned}$$

where the factors on the right-hand side are all nondimensional. This Eq. (48) is valid as long as the production is non-negative. We tentatively restrict our attention to this case of sufficiently small state fluctuations for simplicity.

Two nondimensional parameters governing the system’s behavior are thus identified: $CE_0^2/\nu ^1$ controls the nondimensional rate at which the target potential is approached. Similarly, $b^1C/\nu ^1$ controls the rate at which the target effective area is approached.

#### Parametric study of stability

We are interested in the stability of the proposed dynamical system subject to changes in the flow conditions, which in turn affect the target state $\hat{\Omega }=({\hat{\mathbf {A}}},\hat{\lambda })$ and $\hat{\Pi }$ of Eq. (48). The parameters, $CE_0^2/\nu ^1$ and $b^1 C / \nu ^1$, of the nondimensional evolution equation could potentially affect the stability. We evaluate the stability for a particular choice of parameters by starting a simulation from a homeostatic state at the point of operation. As a test perturbation, we apply a $1\,\%$ step increment to the target stretch $\hat{\lambda }$ under a constant pressure assumption. Due to the equilibrium equation for the homeostatic case, Eq. (28), this also produces a step change in $\hat{A}^1$. Equation (33) is integrated by a forward Euler method, where convolution integrals are approximated using the trapezoidal rule, as described in Appendix 2.

**Fig. 5:**
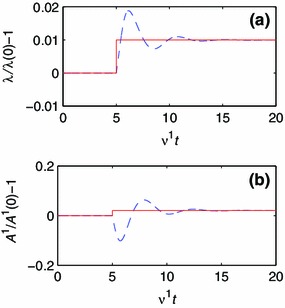
Development of geometry and composition for a one-constituent model with parameters $CE_0^2/\nu ^1 = 10^5$ and $b^1 C / \nu ^1 = 10^{-1}$. The *solid line* represents the target value, and the *dashed line* represents the current value. **a** The development of $\lambda$. **b** The development of $A^1$.

**Fig. 6:**
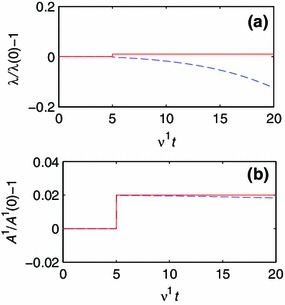
Development of geometry and composition for a one-constituent model with parameters $CE_0^2/\nu ^1 = 10^4$ and $b^1 C / \nu ^1 = 10^2$. The *solid line* represents the target value, and the *dashed line* represents the current value. **a** The development of $\lambda$. **b** The development of $A^1$.

**Fig. 7:**
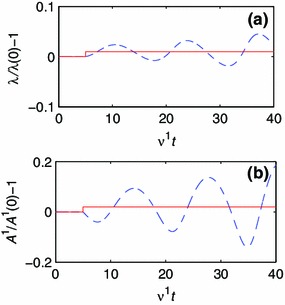
Development of geometry and composition for a one-constituent model with parameters $CE_0^2/ \nu ^1 = 10^4$ and $b^1 C / \nu ^1 = 10^{-2}$. The *solid line* represents the target value, and the *dashed line* represents the current value. **a** The development of $\lambda$. **b** The development of $A^1$.

For each investigated parameter pair, $(CE_0^2/\nu ^1,b^1 C / \nu ^1)$, the simulation results are evaluated by observing the development of $A^1$ and $\lambda$. If either one of these values fail to approach the respective target values, $\hat{A}^1$ and $\hat{\lambda }$, the parameter pair is deemed unstable, otherwise stable. Three qualitatively different responses to the step change in $\hat{\lambda }$ are identified. These are illustrated for the one-constituent, isotropic material model in Figs. 5, 6 and 7.

The development of $\lambda$ and $A^1$ for parameters $CE_0^2/\nu ^1=10^5$ and $b^1 C / \nu ^1=10^{-1}$ are plotted in Fig. 5. When the target stretch increases by $1$ %, the stretch $\lambda$ increases and exhibits overshoot and ringing when approaching the target stretch. The referential area decreases initially, only to later approach the target value. The system’s strategy is thus to initially reduce the referential area and allow the vessel to expand. This behavior is referred to as convergent (Conv.).

In Fig. 6, we study the model response for $CE_0^2/\nu ^1=10^4$ and $b^1 C / \nu ^1=10^2$. In this case, $A^1$ reaches its target value very rapidly, but since the growth of the materials reduces the stretch, $\lambda$ diverges monotonically to zero. We label this behavior monotonic divergence (MD).

Finally, we study the response for $CE_0^2/ \nu ^1= 10^4$ and $b^1 C / \nu ^1=10^{-2}$ (Fig. 7). After the step change in $\hat{\lambda },\lambda$ and $A^1$ oscillate with increasing amplitudes, and $A^1$ lags $\lambda$ by 90 degrees. We call this condition oscillatory divergence (OD).

These three different behaviors, convergence, monotonic divergence and oscillatory divergence, are observed for the one-constituent, orthotropic material model and for the two-constituent model as well.

## Results and discussion

### Stability analyses

**Fig. 8:**
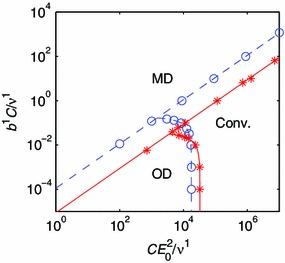
Regimes of convergence and divergence for the one-constituent models. The *dashed line* and *rings* represent an isotropic material model. The*solid line* and *stars* represent an orthotropic material model. The labels indicate the regimes of monotonic divergence (MD), oscillatory divergence (OD) and convergence (Conv.)

By systematically exploring the nondimensional parameter two-space of the evolution equation, the regions corresponding to convergent and divergent model responses are uncovered. The three observed behaviors, Conv., MD and OD, form three simply connected domains in the $(C E_0^2/\nu ^1,b^1 C / \nu ^1)$ plane for the one-constituent, isotropic material model. Points on the boundaries between these domains are identified using the bisection method, and the boundary lines are obtained by interpolation, and also extrapolation for small values of $C E_0^2/\nu ^1$ and $b^1 C / \nu ^1$. These boundary points and inferred boundaries are indicated by rings and dashed lines in Fig. 8.

**Fig. 9:**
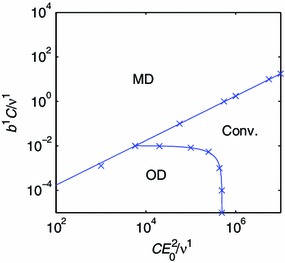
Regimes of convergence and divergence for the two-constituent model. The labels indicate the regimes of monotonic divergence (MD), oscillatory divergence (OD) and convergence (Conv.)

A straight line with slope one in the log–log diagram separates the MD regime from the other two regimes. This shows that MD is observed when the ratio $b^1/E_0^2$ between the nondimensional parameters exceeds some critical value. The remaining regimes, OD and Conv., are separated by a curve that intersects the $C E_0^2/\nu ^1$ axis. This qualitative picture is still standing for the one-constituent, orthotropic material model, shown as stars and a solid line in Fig. 8, and the two-constituent model (Fig. 9). It is thus possible to make observations of some generic properties of the evolution equation.

Consider the extreme case of a goal function that is only controlled by the target composition. In our stability analysis, this corresponds to the case $C E_0^2/\nu ^1 \rightarrow 0$ at a finite $b^1 C / \nu ^1$, for which MD is observed. It is thus impossible to achieve convergence by only taking into account the target composition.

Now, consider the extreme case of a goal function that only includes the potential energy term. This corresponds to $b^1 C / \nu ^1 = 0$. Interestingly, convergence can be achieved by choosing a sufficiently large parameter $C E_0^2/\nu ^1$ in this degenerate case. The potential energy term of the goal function still has a (local) minimum at the target state $(\hat{\lambda },\hat{A}^1)$. Therefore, when there is only one component that remodels, the potential energy term is by itself sufficient for controlling growth.

When there are multiple components that remodel, inspection of the goal function, Eq. (31), implies that the minima are any states that satisfy $\Pi [\lambda (\mathbf {A}),\mathbf {A}] = \hat{\Pi }$. This single scalar equation may have infinitely many solutions for the vector $\mathbf {A}$. Then, a target composition is necessary to ensure uniqueness of the target state.

When both terms of the goal function are included, convergence depends on the ratio $b^1/E_0^2$ between the nondimensional parameters. If this ratio $b^1/E_0^2$ is too large, the goal function’s minimum at $(\hat{\lambda },\hat{A}^1)$ is lost and $\lambda$ would drift while the evolution equation maintains an approximately constant $A^1 = \hat{A}^1$ (Fig. 6). This situation occurs when the composition term of the goal function becomes too influential. If $b^1/E_0^2$ is sufficiently small to prevent MD, OD may still occur if $CE_0^2 / \nu ^1$ is too small, which is associated with the production rate of materials in response to the potential energy term being too small.

The active control of the vessel diameter by VSM to maintain a homeostatic wall shear stress is not included in the model, and previous investigations indicate that this active control has a stabilizing effect on growth (Taber 1998). The regime of convergence seen in Fig. 9 is thus likely to be a subset of the true regime of convergence, and our results should be understood as being qualitative. The authors intend to augment the model with an active VSM constituent in future publications.

### Effects of material properties and composition

**Fig. 10:**
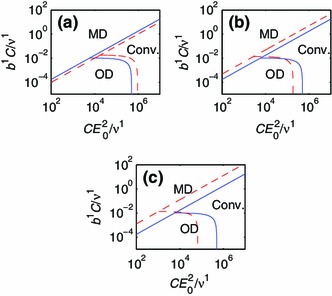
Effects of changes in the material properties. The *solid lines* in **a**, **b** and **c** represent the two-constituent model for a normal vessel. The *dashed line* in (a) represents a vessel without elastin. The *dashed line* in **b** represents a vessel with the material parameter $c_1^1$ increased by factor 2. The *dashed line* in **c** represents a vessel with the material parameter $c_2^1$ increased by factor 1.2.

As people age, the elastin concentration of some blood vessels, such as the ascending thoracic aorta, is reduced (Tsamis et al. 2013). Under pathological conditions, for instance AAA (Choke et al. 2005; Tsamis et al. 2013), the degradation of elastin occurs faster, leading to reduced elastin concentration (Choke et al. 2005; Tsamis et al. 2013). It is an interesting and important question whether this elastin degradation can lead to growth instability and thinning of the vessel wall. For instance, in the theoretical work of Alford and Taber (2008), it is demonstrated that a constituent with slow turnover (elastin) is needed to prevent a developing artery from growing longitudinally without bounds. We conduct a stability analysis for the two-constituent material model with $\phi ^2 = 0$ and $\phi ^1=1$, corresponding to a case of vanishing elastin concentration. The material parameters of the anisotropic constituent, $k=1$, are unchanged (Table 1). The domains of convergence for the evolution equation of a normal and an elastin-deficient vessel, respectively, are compared in Fig. 10a.

We observe that the region of convergence is smaller for the vessel without any isotropic material component. This suggests that the growth of a blood vessel with a reduced amount of elastin has an increased risk of becoming unstable.

The stiffness of the blood vessels increases with age (Nyhan et al. 2011; Lee and Park 2013). It is not clear whether increased stiffness of the vessel wall causes disease. However, there is a clear association between atherosclerosis and vessel stiffness (Popele et al. 2001). We model this by increasing the material parameter $c_1^1$, which is proportional to the strain energy of the anisotropic constituent. Particularly, we increase $c_1^1$ of the two-constituent model by a factor 2, while keeping all other parameters the same as in a healthy vessel (see Table 1). A stability analysis is then carried out, as previously described. From this stability analysis, we see that the domain of convergence increases for the vessel of increased stiffness (see Fig. 10b). This suggests that the growth process of the blood vessels becomes more stable when the constituents stiffen.

The other parameter of the anisotropic material, $c_2^1$, is also a stiffness parameter in the sense that the derivative of the strain energy increases with $c_2^1$. Therefore, we also perform a stability analysis where the two-constituent model parameter $c_2^1$ is increased by a factor 1.2. As expected, it is observed that the effect of increasing $c_2^1$ is similar to that of increasing $c_1^1$: The convergent region increases (see Fig. 10c). An increase in the parameter $c_2^1$ thus promotes a more stable growth process.

Our model suggests that elastin-deficiency impairs growth stability and that increased vessel wall stiffness improves stability. Crucially, degradation of elastin and stiffening of the vessel wall correlate in the aging artery. Our interpretation is that the reduced growth instability of the elastin-deficiency is compensated for by the body with the enhanced stability of a stiffer vessel wall. This hypothesis is further supported by mouse models (Cheng et al. 2013), where mice with an elastin-deficiency from birth develop a vascular system with increased wall stiffness and blood pressure, but otherwise normal function.

### Growth instability-related disease states

How fast a biological constituent is degraded depends on the efficiency of the biological catalysts (enzymes) that produce and degrade the constituent (Campbell et al. 1999 pp. 91-94). Elastin is degraded by elastases, whereas collagen is degraded by collagenases (Choke et al. 2005). Many elastases and collagenases belong to the enzyme class Matrix Metalloproteinases (MMPs) (Choke et al. 2005). Inflammation occurs frequently in AAA and similar states. It is unclear whether inflammation is the cause or the side effect of such conditions. Possibly, inflammation causes an expression of enzymes that degrade collagen (Steinmetz et al. 2003). See Choke et al. (2005) for a review. We have already touched upon the effect of elastin-deficiency, which impairs growth stability. The rate of collagen degradation also has a direct effect on growth stability, owing to the occurrence of the rate constant of degradation $\nu ^1$ in the parameters of the evolution equation. If we picture a convergent system represented by a point in the parameter two-space of Fig. 9, an increase in $\nu ^1$ would move that point down and to the left, potentially inducing OD. This suggests that inflammation, and other disease states connected to collagenase and MMP activity, could increase the risk for growth instabilities in the vascular system.

The other rate parameters of the evolution equation, $b^k$ and $C$, are related to the growth signaling and growth capacity. Although phenomenological, the parameter $C$ can be interpreted as a rate factor particular to the growth control system. An increase in $C$ corresponds to a faster response of the growth control. Considering the parameter two-space of Fig. 9, a convergent system would approach the OD regime if the growth control response time increased. Moreover, the presence of the incremental modulus in one of the parameters $CE_0^2/ \nu ^1$ indicates that the stiffness of the constituents will affect stability. A stiffening of the vessel wall promotes a stable growth process (Fig. 9).

An important observation is that blood pressure and flow rate have no direct impact on stability. This can be seen by the fact that the parameters, $C E_0^2/\nu ^1$ and $b^1 C/\nu ^1$, contain neither the pressure nor the volumetric flow. We believe that this is an important feature which allows the growth control to function purposefully for a wide range flow conditions.

## Conclusions

In this paper, we consider the blood vessel to be a thin-walled, cylindrical tube and assume that the load-bearing constituents deform together, but have individual material properties. The growth dynamics are described by modeling the evolution of the effective cross-sectional area of each constituent. A change in effective area follows the direction of steepest descent of a goal function, formulated in such a way that the constituents grow toward a target potential energy and a target composition, representing a homeostatic state.

One purpose of this study is to investigate the stability of the growth process. To this end, we have studied the growth dynamics using three different material models: one simplistic and then two more realistic models. The first model is a one-constituent, isotropic material model, the second is a one-constituent, orthotropic material model, and the third is a two-constituent model with one isotropic material that does not grow or degrade (elastin) and one anisotropic material that degrades and grows. Simulations show that the conditions for stability are qualitatively similar for these three models. Stability can be achieved if the goal function is based only on potential energy, or on both potential energy and composition.

We identified and analyzed two nondimensional parameters, $CE_0^2/\nu ^1$ and $b^1C/\nu ^1$, which control the growth response toward the target potential energy and the target composition, respectively. The two-space formed by these two parameters includes one domain of growth stability and two domains of growth instability. This stability map was subsequently used to identify the mechanisms that can potentially lead to growth instability and related diseases. A stiffening of the vessel wall promotes a stable growth process. It is also concluded that a general reduction in the production rate, as captured by a reduction of our parameter $C$, increases the risk for growth instability. The rate constant of collagen degradation, $\nu ^1$, has a direct effect on growth stability: An increased value of $\nu ^1$ yields a more unstable growth process. In the case of aneurysms, there is experimental evidence that the turnover of collagen increases (Satta et al. 1995; Abdul-Hussien et al. 2007, that is both the rate of degradation and the rate of production of collagen increases in the aneurysm. It is corroborated by our simulation results that an increased turnover could potentially create an unstable growth process in blood vessels.

We also investigated how the material properties and composition affect the stability of the blood vessel, with the intent to relate the model to disease states. This was done by modifying the two-constituent material model. An elastin-deficient vessel was investigated, exhibiting an increased risk for growth instability. In addition, a vessel composed of stiffer materials was investigated, showing that a stiffer material yields a more stable growth process.

## Appendix A: Time-averaging

To separate high- and low-frequency processes, we define the time window-average of a quantity $y$ by

49 $$\begin{aligned} \langle y \rangle _T = \frac{1}{T} \int \limits _{t-T/2}^{t+T/2} y(\tau ) d \tau . \end{aligned}$$

Generally, $\langle y \rangle _T$ depends on both the time window $T$ and the time $t$.

Let $y_0(t)$ be a function that varies at timescales much greater than $T$, and let $\tilde{y}(t)$ be a function that only varies at timescales much smaller than $T$, with $\langle \tilde{y}\rangle _T = 0$. Then, because $y_0$ can be treated as a constant within each time window to a good approximation, it follows directly from the definition of the time-average that

50 $$\begin{aligned} \langle y_0\rangle _T(t) = \frac{1}{T} \int \limits _{t-T/2}^{t+T/2} y_0(\tau ) d \tau = \frac{1}{T} \int \limits _{t-T/2}^{t+T/2} y_0(t) d \tau = y_0(t).\nonumber \\ \end{aligned}$$

There is no $T$-dependence for slowly or rapidly varying functions, and we may thus drop the index $T$, denoting the time window-average by $\langle \cdot \rangle$.

Now, if we construct a function $y(t) = y_0(t) + \tilde{y}(t)$, we find that $\langle y \rangle = \langle y_0 \rangle + \langle \tilde{y}\rangle = y_0$, so that

51 $$\begin{aligned} y = \langle y \rangle +\tilde{y}. \end{aligned}$$

Moreover, for any function $g$ with continuous derivatives, letting ‘$\circ$’ denote function composition, we have

52 $$\begin{aligned} \langle g \circ y \rangle (t)&= \frac{1}{T}\int \limits _{t-T/2}^{t+T/2} g[y_0(\tau ) + \tilde{y}(\tau )] d \tau \nonumber \\&= \frac{1}{T} \int \limits _{t-T/2}^{t+T/2} g[y_0(t)+ \tilde{y}(\tau )] d \tau \nonumber \\&= \frac{1}{T} \int \limits _{t-T/2}^{t+T/2} \left\{ g[y_0(t)] + d g[y_0(t)]\tilde{y}(\tau )\right. \nonumber \\&\quad \left. + \sum _{n=2}^\infty \frac{d^n g[y_0(t)]}{n!}[\tilde{y}(\tau )]^n \right\} d \tau \nonumber \\&= (g \circ y_0)(t) + (dg \circ y_0)(t) \underbrace{\langle \tilde{y} \rangle (t)}_{=0}\nonumber \\&\quad + \sum _{n=2}^\infty \frac{1}{n!}(d^n g \circ y_0)(t) \langle \tilde{y}^n \rangle (t) \nonumber \\&= (g \circ y_0)(t) + \sum _{n=2}^\infty \frac{1}{n!}(d^n g \circ y_0)(t) \langle \tilde{y}^n\rangle (t).\nonumber \\ \end{aligned}$$

Hence, using that $y_0 = \langle y_0 \rangle = \langle y \rangle$ in Eq. (52), a first-order approximation yields

53 $$\begin{aligned} \langle g \circ y \rangle \approx g \circ \langle y \rangle , \end{aligned}$$

and the approximation becomes an identity for slowly varying functions, *i.e.,* when $\tilde{y}=0$.

## Appendix B: Numerical scheme

### B.1 Age distribution of materials

We introduce the age distribution of materials

54 $$\begin{aligned} \alpha ^k(t,\tau ) = \fancyscript{A}^k(\tau )q^k(t-\tau ),\quad t \ge 0, \quad 0 \le \tau \le t. \end{aligned}$$

Then $\alpha ^k(t,\tau ) d\tau$ represents the amount of constituent $k$ that remains at time $t$ and was produced within the time interval $[\tau ,\tau + d\tau )$. From this definition, it follows directly that

55 $$\begin{aligned} \alpha ^k(t + \Delta t,\tau ) = \frac{q^k(t + \Delta t -\tau )}{q^k(t-\tau )} \alpha ^k(t,\tau ) \end{aligned}$$

for any time difference $\Delta t$. It also follows that

56 $$\begin{aligned} \alpha ^k(t,t) = \fancyscript{A}^k(t)q^k(0) \quad \Leftrightarrow \quad \fancyscript{A}^k(t) = \alpha ^k(t,t). \end{aligned}$$

Time is discretized into a set of equidistant points

57 $$\begin{aligned} t_n = n \Delta t, \quad n=0,1,2,\ldots ,N, \end{aligned}$$

with time-step $\Delta t$. Then, $\alpha ^k$ is discretized as

58 $$\begin{aligned} \alpha ^k_{n,j} = \alpha ^k(n\Delta t, j\Delta t),\quad j =0,\ldots ,n. \end{aligned}$$

According to Eq. (55), without any approximation, we have

59 $$\begin{aligned} \alpha ^k_{n+1,j} = c^k_{n,j} \alpha ^k_{n,j},\quad c^k_{n,j} =\frac{q^k[(n-j+1)\Delta t]}{q^k[(n-j)\Delta t]}. \end{aligned}$$

We now approximate the integral of Eq. (18) by a sum, thus obtaining

60 $$\begin{aligned} A^k_n = A^k_0 Q^k(n \Delta t) + \left[ \frac{\alpha ^k_{n,0} + \alpha ^k_{n,n}}{2} + \sum _{j=1}^{n-1} \alpha ^k_{n,j}\right] \Delta t,\nonumber \\ \quad n > 0. \end{aligned}$$

Hence, the increment of $A^k$ between two time steps can, without further approximation, be expressed as

61 $$\begin{aligned} \frac{A^k_{n+1} - A^k_n}{\Delta t}&= A^k_0\frac{Q^k(n \Delta t + \Delta t)-Q^k(n \Delta t)}{\Delta t} +\nonumber \\&\quad \frac{\alpha ^k_{n+1,0} + \alpha ^k_{n+1,n+1}}{2} - \frac{\alpha ^k_{n,0} + \alpha ^k_{n,n}}{2}\nonumber \\&\quad + \sum _{j=1}^{n} \alpha ^k_{n+1,j} - \sum _{j=1}^{n-1} \alpha ^k_{n,j} \nonumber \\&= A^k_0\frac{Q^k(n \Delta t + \Delta t)-Q^k(n \Delta t)}{\Delta t} + \nonumber \\&\quad \frac{c^k_{n,0}\alpha ^k_{n,0} + \alpha ^k_{n+1,n+1}}{2} - \frac{\alpha ^k_{n,0} + \alpha ^k_{n,n}}{2}\nonumber \\&\quad + \sum _{j=1}^{n} c^k_{n,j}\alpha ^k_{n,j} - \sum _{j=1}^{n-1} \alpha ^k_{n,j} \nonumber \\&= A^k_0\frac{Q^k(n \Delta t + \Delta t)-Q^k(n \Delta t)}{\Delta t} + \frac{1}{2}\alpha ^k_{n+1,n+1}\nonumber \\&\quad +\,\frac{c^k_{n,0}-1}{2}\alpha ^k_{n,0} + \frac{2c^k_{n,n}-1}{2}\alpha ^k_{n,n}\nonumber \\&\quad +\, \sum _{j=1}^{n-1} (c^k_{n,j}-1) \alpha ^k_{n,j} \end{aligned}$$

### B.2 Time increment iteration

A time increment from time $n \Delta t$ to time $(n+1)\Delta t$ is achieved by following these steps:

1. Compute the new effective areas for all materials using the mid-point formula
  62 $$\begin{aligned} A^k_{n+1} = A^k_{n} - C \Delta t \left. \frac{\partial f}{\partial A^k} \right| _n, \end{aligned}$$
  where the partial derivative $\partial f/\partial A^k$ is evaluated using Eq. (32).
2. For each constituent $k$, compute $\alpha ^k_{n+1,n+1}$ using Eq. (61). If the computed $\alpha ^k_{n+1,n+1}$ is negative, we must set $\alpha ^k_{n+1,n+1} = 0$. This is because $\alpha ^k_{n+1,n+1}$, by Eq. (56) is identical to the production at time $(n+1)\Delta t$, which cannot be negative. Because of this correction, $A^k_{n+1}$ must also be recomputed using Eq. (61) with $\alpha ^k_{n+1,n+1} = 0$.
3. Compute $\alpha ^k_{n+1,j},j = 0,1,\ldots ,n$ for each constituent $k$ using Eq. (59).
4. The strain energy per unit length at time $(n+1)\Delta t$ is obtained from Eq. (21):
  63 $$\begin{aligned} A^{k}_{n+1}\psi ^{k}(\lambda )&= A^{k}_0 Q^{k}(n\Delta t+ \Delta t) \Psi ^{k}\left( \frac{\lambda }{\lambda _0}G^k_{\mathrm {h}}\right) + \nonumber \\&\frac{\Delta t}{2}\left[ \alpha ^k_{n+1,0} \Psi ^{k}\left( \frac{\lambda }{\lambda _0}G^k_{\mathrm {h}} \right) + \alpha ^k_{n+1,n+1} \Psi ^{k}\right. \nonumber \\&\times \left. \left( \frac{\lambda }{\lambda _{n+1}}G^k_{\mathrm {h}} \right) \right] + \nonumber \\&\sum _{j=1}^{n} \alpha ^k_{n+1,j} \Psi ^{k}\left( \frac{\lambda }{\lambda _j}G^k_{\mathrm {h}} \right) \Delta t. \end{aligned}$$
  Differentiate this equation with respect to $\lambda$ and solve the equilibrium Eq. (17) for $\lambda _{n+1}=\left\langle \lambda \right\rangle (n\Delta t+ \Delta t)$; the pressure $\langle p \rangle (n\Delta t+ \Delta t)$ is presumed to be given.
5. Compute the strain energy densities $\psi ^k_{n+1} = \psi ^{k}(\lambda _{n+1})$ for each constituent $k$ using Eq. (63).
6. Compute the potential energy function $\Pi _{n+1} = \Pi (\lambda _{n+1}, \mathbf {A}_{n+1})$ using Eq. (30).

With these operations, all variables have been obtained for the time $(n+1)\Delta t$.
